# Characterization of a mock up nuclear waste package using energy resolved MeV neutron analysis

**DOI:** 10.1038/s41598-025-89879-0

**Published:** 2025-02-25

**Authors:** Tim T. Jäger, Tsviki Y. Hirsh, Stefan Scheuren, Alexander M. Long, Adrian S. Losko, Alexander Wolfertz, Marc Zimmer, Markus Roth, Sven C. Vogel

**Affiliations:** 1 Institut für Kernphysik, Technische Universität Darmstadt, Darmstadt, Germany; 2 Materials and Science Division, Los Alamos National Laboratory, Los Alamos, USA; 3 Soreq Nuclear Research Center (SNRC), Yavne, Israel; 4 Forschungs-Neutronenquelle Heinz Maier-Leibnitz (FRM II), Technische Universität München, Munich, Germany; 5 Focused Energy GmbH, Darmstadt, Germany

**Keywords:** Energy science and technology, Nuclear energy, Nuclear waste

## Abstract

Reliable radiographic methods for characterizing nuclear waste packages non-destructively (without the need to open containers) have the potential to significantly contribute to safe handling and future disposal options, particularly for legacy waste of unknown content. Due to required shielding of waste containers and the need to characterize materials consisting of light elements, X-ray methods are not suitable. Here, energy-resolved MeV neutron radiography is demonstrated as a first-of-its-kind application for non-destructive and remote examination of mock up nuclear waste packages from a safe position using time-of-flight techniques enabled by a novel event-mode imaging detector system. Energy-resolved neutron transmission spectra were measured spatially, permitting the detection of analogue materials to actual nuclear waste such as water, melamine, and ion exchange resin within a 2.54 cm wall thickness steel pipe. The results demonstrate the capability to locate the materials through this wall thickness by radiography and tomographic reconstruction, revealing detailed 3D distributions and structural anomalies. The method effectively detects residual water in ion exchange resin, highlighting its sensitivity to moisture content, a crucial parameter for nuclear waste characterization. Monte Carlo simulations are in agreement with the experimental findings, providing a pathway to simulate waste forms more difficult to tackle experimentally. This work paves the way to apply sub-nanosecond intense MeV neutron sources, such as laser-driven neutron sources under development, to nuclear waste characterization.

## Introduction

The management of nuclear waste presents significant challenges worldwide, particularly in countries relying on nuclear power for a substantial fraction of their electric power production where considerable amounts of nuclear waste are stored, often in aging facilities with significant amounts of legacy waste requiring characterization^1,2^. The urgency to address these challenges is underscored by the need for safer storage, transportation, and eventual disposal of nuclear waste, especially as the timeline for establishing geological disposal facilities approaches^1,3^. For final storage in a repository, each nuclear waste container needs to be characterized according to national regulations and defined waste acceptance criteria of the disposal facilities^4^. This includes documentation of the radioactive inventory as well as documentation of the non-radioactive but often toxic or hazardous content^5^. In Germany, the regulations also do not allow the presence of liquids or overpressure inside the containers and the structural integrity of the containers needs to be verified^2^. Legacy waste, in particular, poses a significant challenge due to the unknown nature of contributing waste streams because of non-existent or incomplete documentation^6^.

For low-level waste, common practices are the manual inspection of undocumented waste, causing a health hazard to workers, and frequent core drillings to statistically verify existing documentation^7^. For intermediate-level waste, inspection is particularly challenging due to the high dose rate that imposes expensive robotic systems. After the inspection, the waste must be reconditioned and repackaged into new containers, increasing the overall cost^4,7–10^. Although many legacy containers may ultimately require repackaging to meet modern safety standards, e.g. Bestandsschutz (= grandfathering) regulations in Germany allow some containers to remain in their original form if the unknown content has been adequately characterized. Furthermore, even when repackaging is necessary, non-destructive methods are useful for material identification before opening the waste package, to enable safe handling. Therefore, radiographic methods can contribute to minimizing dose for workers as well as safe, reliable, and cost-efficient handling of nuclear waste of all waste levels, including high-level waste^11^.

**Figure 1:**
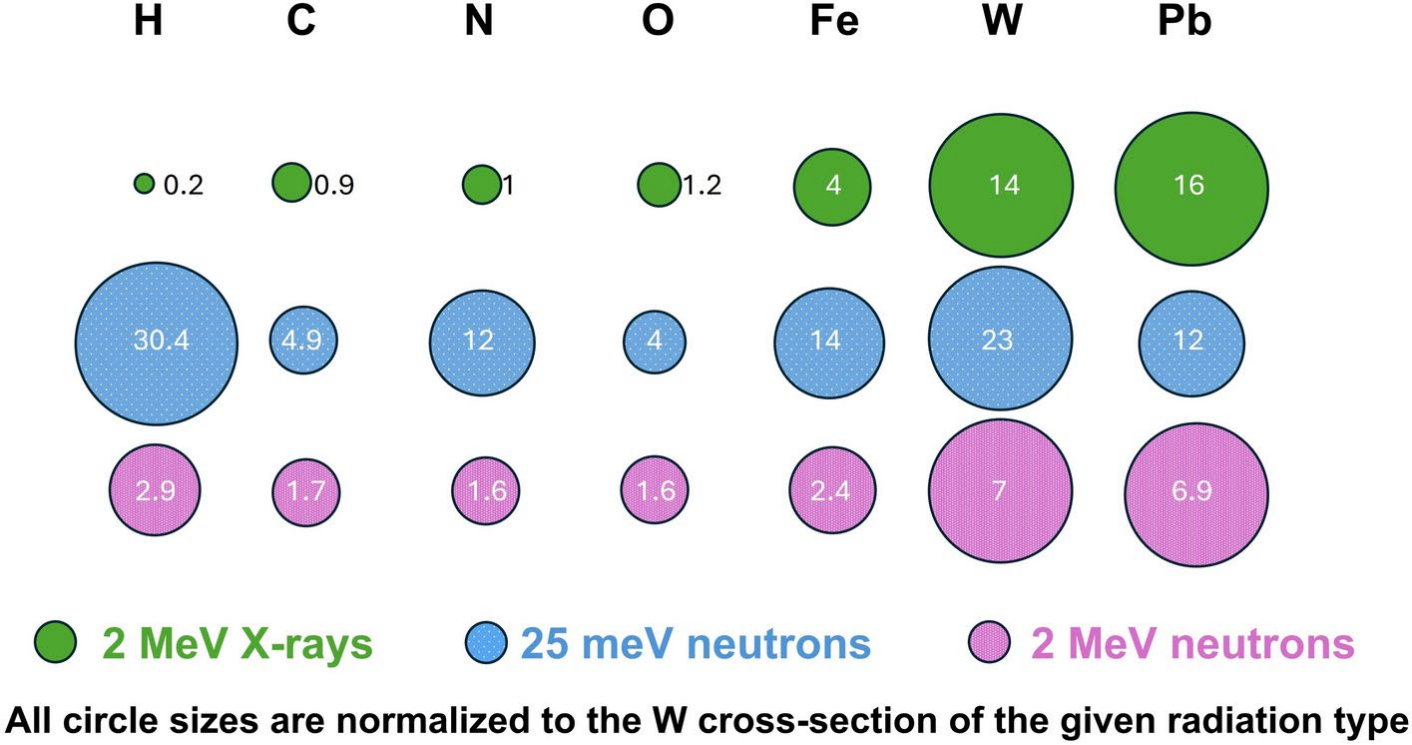
Schematic of X-ray, thermal-, and fast neutron total cross-sections for different elements. The cross-sections are given in b. The areas of the circles are proportional to the corresponding cross-sections, but for every radiation type circle sizes are normalized to their tungsten cross-section. MeV neutrons show the least variation in cross-sections and therefore uniquely enable characterization of high and low-Z elements simultaneously. Neutron Data taken from^25^ and X-ray data from^26^.

Multiple methods for non-destructive nuclear waste inspection have been explored, ranging from X-ray radiography^12,13^ to neutron activation analysis^14^ to ultrasonic inspection^15^. MeV neutron radiography and tomography have been especially promising as high energy neutrons have a deep penetration depth and can propagate through high-Z shielding materials that may be in place to stop the gamma radiation emerging from the radioactive waste. Thermal neutrons and X-rays would be severely attenuated by several centimeters of steel. Figure 1 shows the total cross-sections of X-rays, thermal-, and MeV neutrons for some elements of interest, illustrating that for MeV neutrons high- and low-Z materials have comparable cross-sections while the cross-sections for shielding materials such as Fe and Pb are sufficiently low to enable penetration into shielded containers^16^. Furthermore, compact ($<\,15\,\times \,{15}\,{\hbox {m}}^{2}$) and semi-mobile neutron sources such as laser-driven neutron sources, typically produce MeV neutrons originating from a small source size in the direction of the laser pulse^17–19^, optimal for magnified energy-resolved MeV neutron imaging applications. Similar accelerator-based compact neutron sources, which are 5 m in length, and weigh 6 t including shielding, have been developed recently^20^. They deliver $10^{11}$ n/s and are currently being optimized to fit on a truck^21^. While their pulse width is with $>\,{1}\,\upmu {\hbox {s}}$^20^ not suitable for MeV neutron time-of-flight techniques, commercially available laser systems capable of driving short-pulse X-ray and neutron sources are currently under development^22–24^. Moderation for thermal neutrons would distribute the directed neutron pulses over $4\pi$ at a significant loss of flux of the neutrons available for imaging. Avoiding moderation is therefore beneficial also from a flux and source size perspective. Moreover, MeV neutrons have a strong sensitivity for low-Z materials like hydrogen, carbon, or oxygen, which are especially hard to detect with X-ray sources behind the shielding materials.

Neutron imaging of nuclear waste can pose some safety considerations, such as potential activation and criticality risks. However, most materials susceptible to activation are already activated from reactor operations, limiting additional activation concerns. Furthermore, it is worth pointing out that the amount of neutrons interacting with the sample during such a characterization, even with hours of exposure, is many orders of magnitude away from the amount of atoms in a sample and therefore does not produce any significant amount of fission products or change the isotopic composition in general. Additionally, criticality risks can be managed by active interrogation via a single-shot neutron scan (e.g., with a laser-driven source), which may be used to detect fissile material through analysis of the delayed neutron output, thus allowing to identify waste packages posing a criticality risk beforehand^27^.

Recent advancements in neutron radiography have shown that nuclear waste inspection with strong neutron sources is possible if they provide sufficiently high neutron energies and they are equipped with a proper beam collimation system^16^. A downside in previous validation experiments was that these measurements were performed with continuous beam neutron sources or long neutron pulses, prohibiting the use of time-of-flight methods for differentiation of neutron energies. The ability to resolve incident neutron energies directly enables the possibility to perform neutron resonance imaging and spectroscopy in addition to a white beam radiography, allowing to identify materials from their energy-dependent attenuation. This approach, originally developed for identifying explosives by exploiting MeV neutron resonances of light elements, demonstrates the versatility of energy-resolved neutron techniques in material identification^28^.

Recent progress in detector technology, providing so-called event-mode imaging, greatly enhances the signal-to-noise ratio in neutron imaging, and enables efficient detection of neutrons with ns temporal resolution^29^. Furthermore, novel scintillator materials for MeV neutrons, so-called nanoguides^30^, greatly improve data quality. Combined with the event-mode imaging they make nuclear waste characterization with energy-resolved MeV neutron radiography a realistic opportunity.

Building on these advances, this paper explores the use of energy-resolved MeV neutron techniques for the characterization of a mock up nuclear waste package. Utilizing time-of-flight methods enabled by short-pulse spallation neutrons and the unique event-mode imaging detector system, the neutron transmission was measured not just spatially, but also energy-resolved. The Weapons Neutron Research (WNR)^31^ facility at the Los Alamos Neutron Science Center (LANSCE)^32^ is a spallation neutron source providing the required ${\sim }{1}\,{\hbox {ns}}$ short neutron pulses. To the best of our knowledge, this is the first demonstration of spatially-resolved energy-resolved MeV neutron analysis of a (mock up) nuclear waste package. The energy resolution allowed the detection and mapping of water, simulating residual liquid or moist materials, melamine, simulating toxic content, and ion exchange resin, simulating filter materials, based on their individual energy-dependent transmission profiles. This was achieved by fitting their transmission spectra with theoretical transmissions calculated from JENDL4.0^33^ total cross-sections based on the known chemical composition of the materials. In the nuclear power industry, ion exchange resin is often utilized for filtering radioactive contaminants out of liquid waste streams^34^, and for the disposal of this spent ion exchange resin it is crucial to know if any residual water is left in the resin^34–36^. Therefore, ion exchange resin samples containing different amounts of water have been analyzed to determine if residual water or moisture levels can be detected using MeV neutrons. Furthermore, a neutron tomography of the waste drum has been performed to reconstruct the 3D distribution of materials inside the drum, showing that the content can be determined without having to open the waste package. Monte-Carlo simulations have been conducted using the neutron transport code *PHITS*^37^ to compare with the experimental data to benchmark future simulations of more hazardous materials.

## Methods

### Experimental setup

As a test of the feasibility of energy-resolved MeV neutron radiography and tomography for analyzing nuclear waste drums, a mock up waste drum sample was measured at the 60R flight path^38^ of WNR. At the WNR, 800 MeV proton pulses with widths on the order of ${\sim 1}\,{\hbox {ns}}$ impinge on a tungsten target. The resulting spallation neutron spectrum provides neutrons from ${\sim 0.1}\,{\hbox {MeV}}$ up to the proton energy of 800 MeV with a peak flux at ${\sim 1}\,{\hbox {MeV}}$^39^. Proton pulses are delivered in macropulses lasting up to ${625}\,\upmu {\hbox {s}}$, each consisting of 348 micropulses of approximately 1 ns duration, spaced ${1.8}\,\upmu {\hbox {s}}$ apart. The time between successive macropulses is typically 8.3 ms.^40^. The detector was set up at a distance of 22.18 m to the spallation target and a 7.62 cm collimation was used. The collimation started at 4.27 m with a diameter of 8.25 cm, resulting together with the sample positioned at 21.98 m in a theoretical L/D = 215 and a collimation blur of 0.9 mm.

**Figure 2:**
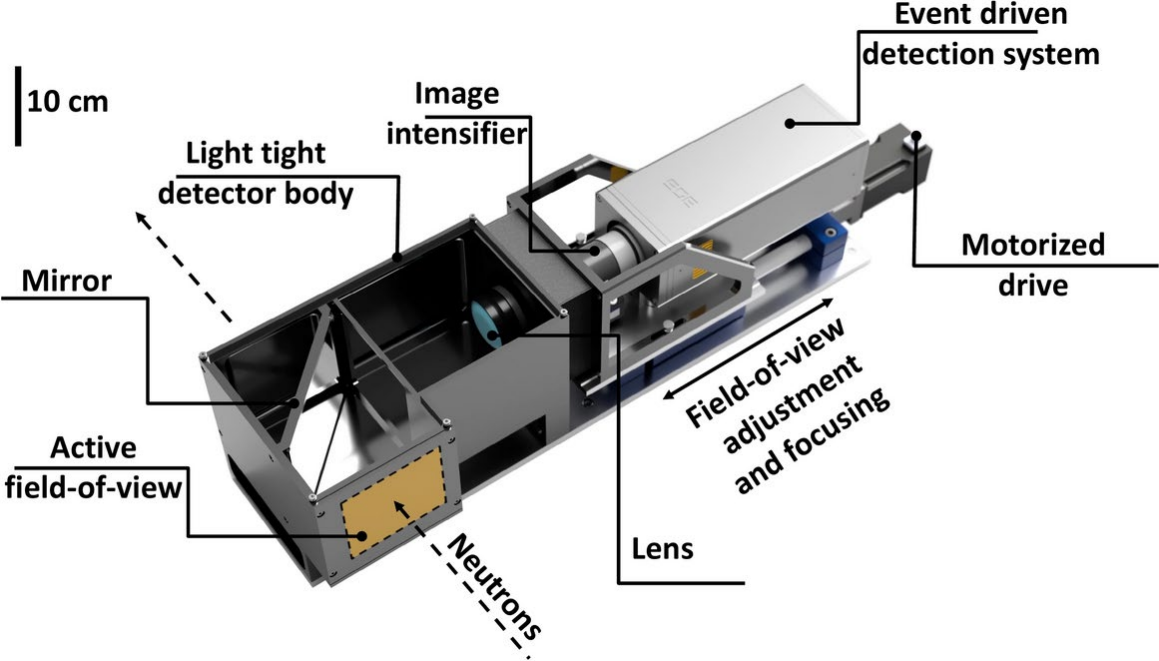
Schematic of the event-mode imaging detector employed in this experiment.

High-energy neutrons are efficiently detected using hydrogen-containing scintillators through elastic reactions with protons (proton recoil). To detect these neutrons effectively, > 1 cm thick scintillators are usually selected. However, the light is emitted along the path of the recoil protons and can extend far from the original neutron interaction point (e.g. mean free path of a 3 MeV proton in the standard EJ200^41^ scintillator is ${141}\,\upmu {\hbox {m}}$^42^). Furthermore, the light is emitted in ${4}\,{\pi }$ and can therefore spread significantly in a thick scintillator before reaching the surface. Both effects cause blurring of the final image, which prevents achieving similar spatial resolution compared to traditional thermal and epi-thermal neutron detection methods. To address these problems in MeV neutron imaging, recent studies have demonstrated the benefits of identifying neutron interactions by analyzing the time-resolved photons emitted from the scintillator due to the neutron interaction, so-called event-mode imaging^29,43^. The LumaCam event-mode imaging system employed in this work, which is depicted in Fig. 2, enables sub-pixel resolution imaging through center-of-gravity algorithms. It also enhances the temporal resolution because the time-of-arrival of the first photon of a neutron event is taken as the event’s time-of-arrival, neglecting the influence of photons produced by the same neutron, but emitted later (e.g. due to scintillator decay). Additionally, this approach allows for better discrimination between neutrons, gammas, and background (e.g. spurious photon emissions from the image intensifier), improving the overall accuracy and effectiveness of the imaging system. The neutron-sensitive region of the detector comprised a $10.2\,\times \,{10.2}\,{\hbox {cm}^{2}}$ scintillator screen consisting of a 2.54 cm thick organic glass nanoguide scintillator faceplate^30^ to have a high detection probability for MeV neutrons. The nanoguide’s unique fiber structure along the beam direction enhances image resolution by minimizing the light spread perpendicular to the beam within the scintillator, preferably constraining scintillation light propagation in one dimension, therefore providing a sharp, well-defined image of the neutron interaction point and associated proton recoil. Employing a $45^{\circ }$ mirror and an optical lens (Navitar, DO-2595^44^, 25 mm focal length, F/0.95), the light emitted from the scintillator screen was focused onto a dual multi-channel plate image intensifier in Chevron configuration (Photonis, $\hbox {Cricket}^{\hbox {TM}2}$ $\hbox {Hi-QE}^{\hbox {TM}}$ Green^45^, 5$\times \,10^5$ gain, P47 phosphor). The intensified light emitted from the back of the image intensifier was read out with a Timepix3-based event-mode camera (Amsterdam Scientific Instruments, TPX3Cam^46,47^). All components were encased within a light-tight enclosure designed by LoskoVision GmbH to minimize background from external light.

**Figure 3:**
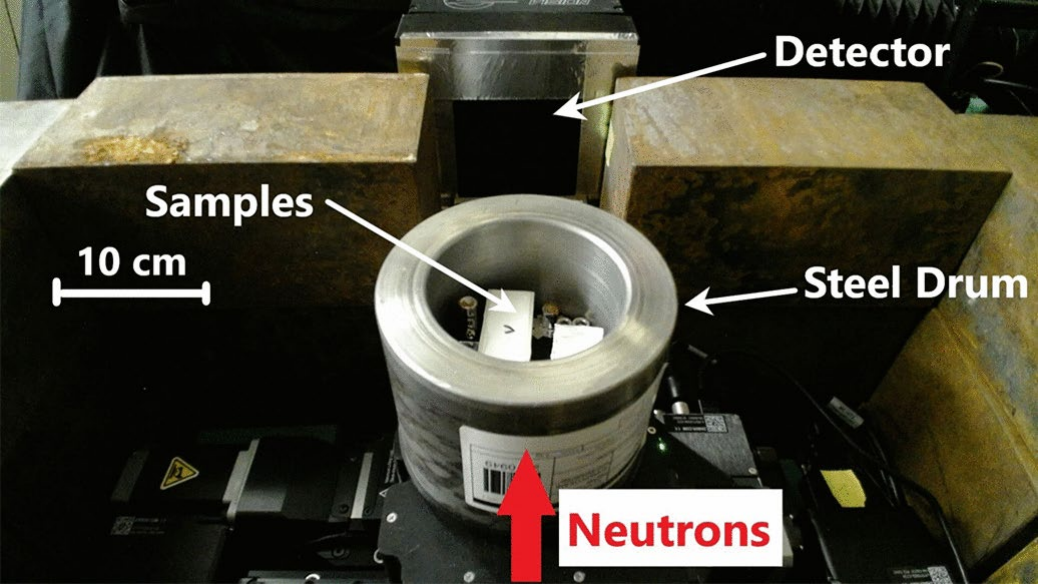
Photograph of the mock up waste drum in front of the neutron detector.

To simulate the several cm thick steel shielding of a nuclear waste drum^48^ realistically, several materials of interest were put into a steel pipe segment with 2.54 cm wall thickness (8.50 cm inner diameter, 13.58 cm outer diameter) as shown in Fig. 3. The thick steel was supposed to simulate the shielding effects of the actual steel drums and heavy elements in nuclear waste packages. The mock up waste drum was mounted onto heavy duty motions stages that include a linear stage (Zaber, LRT0250AL-AE53CT10A^49^) and rotations stage (Zaber, X-RST120AK-DE50^50^) to enable alignment optimizations and the tomography. Inside the steel drum were the following materials:

- M3 hex socket head steel screw, simulating a general object
- M4 hex socket head steel screw, simulating a general object
- A so-called SIMFUEL rodlet consisting of three pellets of an oxide mix of $\hbox {dUO}_2$, $\hbox {La}_2$ $\hbox {O}_3$, $\hbox {ZrO}_2$, $\hbox {RhO}_2$, 5.26 mm diameter, total height of 16.53 mm, simulating a nuclear fuel
- A second SIMFUEL rodlet consisting of a stack of penny sized sintered pellets of $\hbox {dUO}_2$, $\hbox {La}_2$ $\hbox {O}_3$, $\hbox {ZrO}_2$, $\hbox {RhO}_2$, and mixed oxide SIMFUEL, 5.26 mm diameter, total height of 26.98 mm, simulating a nuclear fuel
- $2.00\,\times \,2.00\,\times {2.00}\,{\hbox {cm}^{3}}$ container filled with water, simulating residual liquid
- $2.07\,\times \,2.00\,\times \,{5.00}\,{\hbox {cm}^{3}}$ container filled with 15.1 g melamine ($\hbox {C}_3$ $\hbox {H}_6$ $\hbox {N}_6$), simulating toxic content
- one out of three $2.00\,\times \,2.00\,\times \,{5.00}\,{\hbox {cm}^{3}}$ containers filled with ion exchange resin and water, simulating spent ion exchange resin with different water content:
  - 10.81 g resin and no water
  - 10.88 g resin and 5.56 ml water $\hat{=}$ max. retention capability of resin
  - 11.58 g resin and 2.58 ml water $\hat{=}$ half the max. capability.

**Figure 4:**
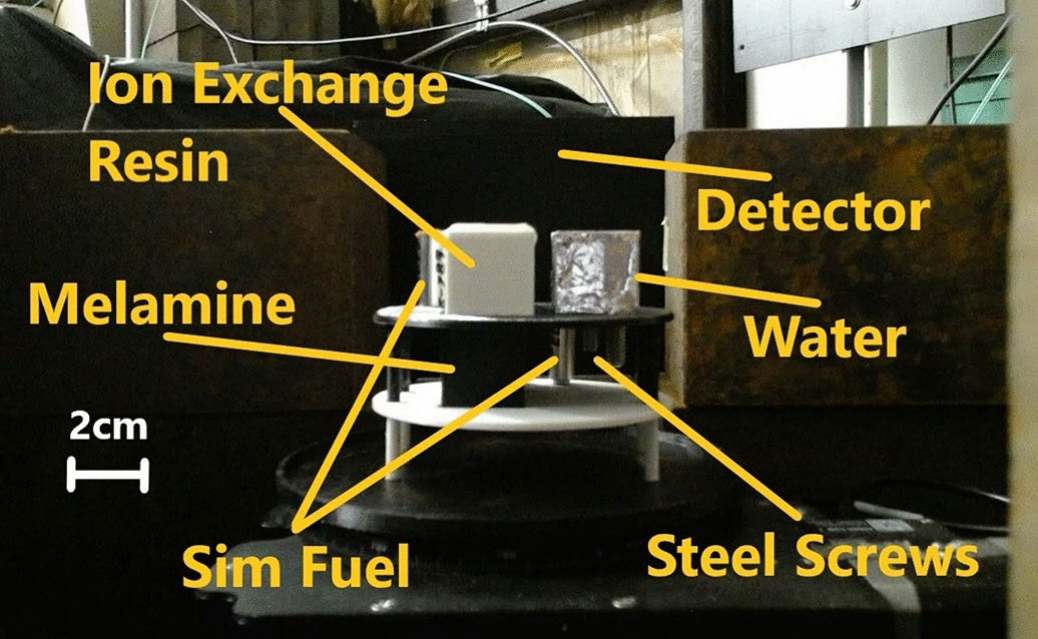
Photograph of the sample without the steel.

All containers and the material holder floors were 3D-printed from PETG with the dimensions listed above being the inner dimensions. The water and melamine containers were printed with a wall thickness of 1 mm while the resin containers have 1.5 mm thick walls. Since over time water was lost, the resin samples were renewed right before the experiment. All materials were arranged in the steel drum as depicted in Fig. 4.

### Data analysis setup

The LumaCam records a list of pixel hits, capturing the x- and y-coordinates, the time-over-threshold, and the time-of-arrival for each pixel that receives enough light from the image intensifier to exceed a set threshold. From this list the neutron events were reconstructed using the two-step procedure described by Losko and Wolfertz et al.^29,43^. In the first step, the scintillator photons hitting the image intensifier are reconstructed, as every photon incident on the image intensifier results in thousands to millions of photons emitted at its back, thus illuminating several neighboring pixels of the Timepix3 sensor. The second step is reconstructing neutron events by searching for clusters of scintillator photons identified in step 1 and filtering noise such as scintillator afterglow and gammas through application of a cluster size threshold. Finally, the neutron events are binned in space and time and an image stack is produced in which every slice represents all neutrons detected in a certain time-of-flight bin. This data reduction process depends on several parameters that influence the clustering and noise rejection, and therefore have to be selected carefully. The spatial and temporal clustering radii determine which scintillator photons are added to an event, and therefore also the area averaged by the center-of-gravity algorithm. If the radii are chosen too large, scintillator photons may get falsely assigned to neutron events, falsifying the center-of-gravity and disturbing the neutron-noise discrimination. If the radii are too small, photons resulting from the same neutron interaction might not be assigned to the same event, also negatively affecting the center-of-gravity and the event discrimination, with the potential of this neutron to be counted twice (or more). The thresholds for minimum number of pixel hits to be considered a scintillator photon and scintillator photons to be considered a neutron event, respectively, influence the gamma and noise rejection. If chosen too low, noise or gammas may falsely pass as neutrons, while if the thresholds are set too high, neutron events are falsely rejected, decreasing neutron statistics significantly as can be seen in the following section. This data reduction approach, originally developed for thermal neutron imaging^29^, can also be applied to MeV neutron imaging with entirely different scintillation mechanisms thanks to the configurability of the above mentioned parameters. Except for the scintillator, the detector system remains the same for thermal and MeV neutron imaging, and the difference in scintillators can be accounted for by the adjustable data reduction parameters. This allows data reduction for both neutron energy ranges by the same algorithm, highlighting again the versatility of the event-mode imaging detection approach. Because of the strong dependence on the reduction parameters, in this work a parameter study has been conducted to find the optimal parameters for the setup used in this experiment. The reduction parameters that were varied and the ranges over which they were varied are:

- minimum # pixel hits to be a scintillator photon $k \in \{ 1,2,3 \}$
- minimum # scintillator photons to be a neutron event $m\in \{ 1,2,3 \}$
- spatial clustering radius $p\in \{2.5,3,3.5,4,6,10 \}$ px
- temporal clustering radius $t\in \{ 20,100 \}$ ns.

**Figure 5:**
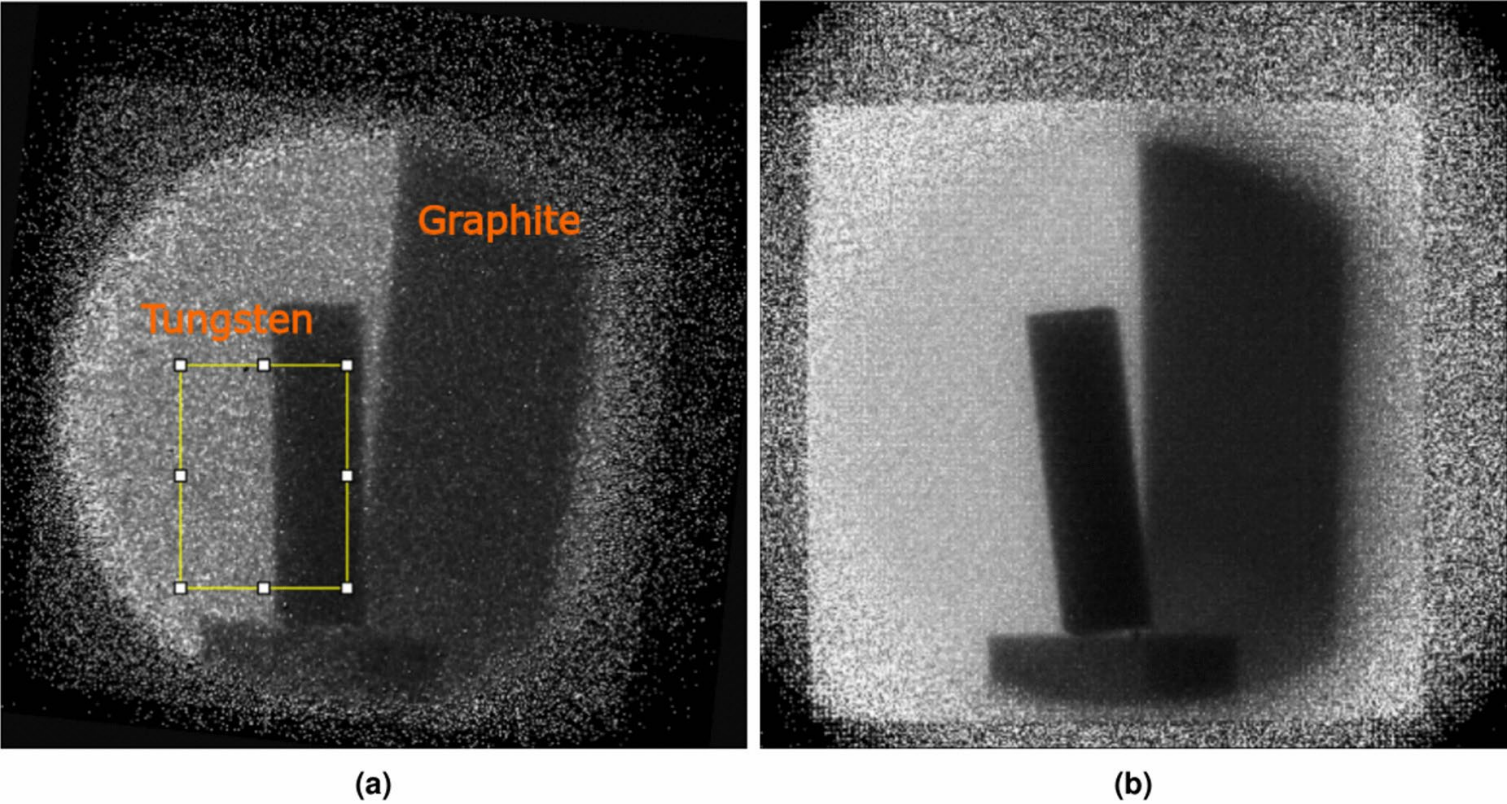
Radiograph of a tungsten and a graphite block used for the parameter scan (neutron energies 2.5 MeV to 8 MeV). Data was evaluated with the highest resolution parameter set (**a**) and the highest contrast parameter set (**b**). (**a**) The yellow box shows the region-of-interest for which the line-spread function and the pixel intensity histogram were determined. The image was rotated clockwise $6^{\circ }$. (**b**) Unlike (**a**) the image has not been rotated.

To identify the best set of parameters all combinations possible with the parameters above were applied to a data set taken within 5 minutes at 60R with the same configurations as described in “Experimental setup”. The sample in front of the detector was a ${1.3}\,{\hbox {cm}}\,\times \,{5.1}\,{\hbox {cm}}\,\times \,{15.2}\,{\hbox {cm}}$ tungsten block and a ${5.1}\,{\hbox {cm}}\,\times \,{10.2}\,{\hbox {cm}}\,\times \,{10.2}\,{\hbox {cm}}$ graphite block. Figure 5a shows a radiograph of this sample made with the parameter-set $k= 1, m = 3, p = 3, t = 20$ and neutron energies 2.5 MeV to 8 MeV.

**Figure 6:**
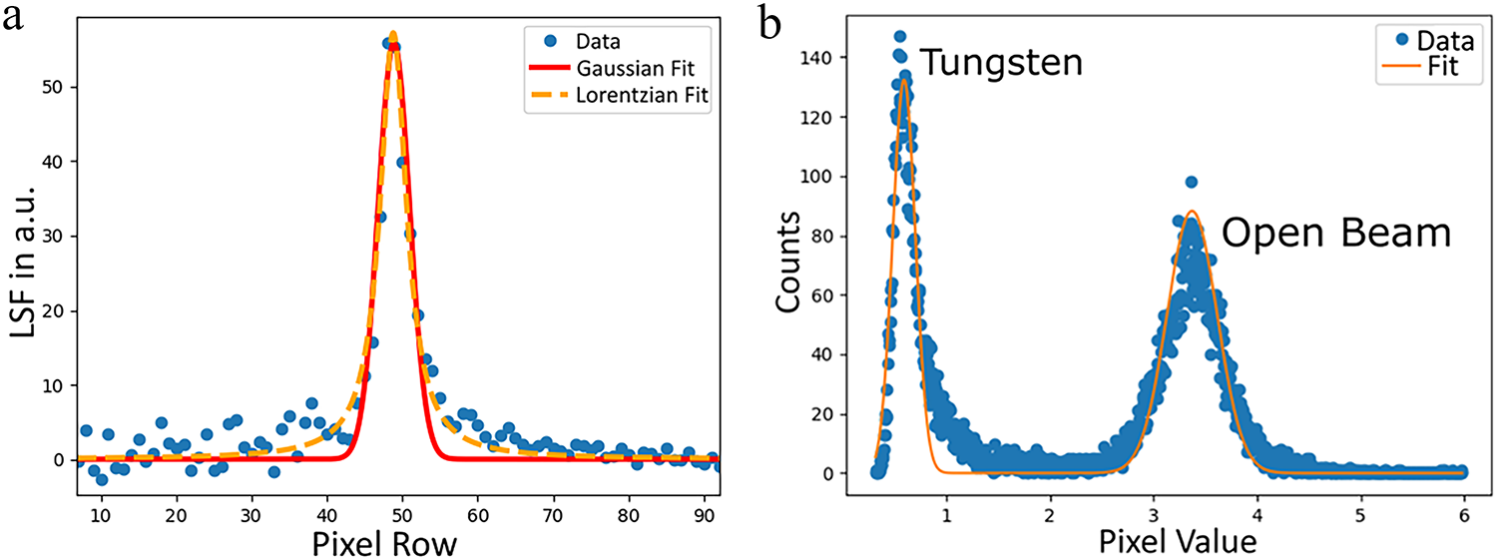
(**a**) Exemplary line-spread-function of the slanted-edge shown in Fig. 5a (but processed with different parameters). The red curve shows a Gaussian fit to the data whereas the orange curve depicts a Lorentzian fit. (**b**) Pixel intensity histogram for the parameter-set showing the best separation (FoM) of the pixel intensities behind the tungsten and for the open beam.

With this neutron energy range a radiograph was produced for every parameter set and every radiograph was corrected by the corresponding flat-field, derived by processing an open beam run with the same parameter set. From the slanted edge in the yellow marked region-of-interest, the resolutions of all radiographs were determined by the full width at half maximum (FWHM) of the line-spread function of the edge. For this, the edge-spread function was first determined by histogramming the neutron counts across the edge and then the line-spread function was determined as its derivative. To quantify the width of the line-spread function, the data points were fitted with a Lorentz function (Eq. 1), as this was found to provide a better match than a Gaussian function (see Fig. 6a).

1 $$\begin{aligned} L(x) = \frac{a \gamma ^2}{ (x-x_0)^2 + \gamma ^2 } \end{aligned}$$

The FWHM of the Lorentzian was calculated as FWHM = $2\gamma$ and taken as the width of the line-spread function, i.e. the resolution of the radiograph. After evaluating all parameter combinations, the parameter set $k= 1, m = 3, p = 3, t = 20$ was found to result in the sharpest edge with a FWHM $={3.62}\,{\hbox {px}}$. This value corresponds to a resolution of 0.9 mm and an experimental L/D=224^51^, in agreement with the values calculated from the beamline geometry in “Experimental setup”. However, as can be seen in Fig. 5a, while this set of parameters produces a sharp edge, the image is noisy due to the reduced neutron counting statistics caused by likely too many neutron events rejected during the data reduction. Since the variation of the resulting resolution across the investigated parameter space was small, but obviously image quality is suffering from excessive rejection of neutron events, all parameter sets were analyzed again for image contrast. For this purpose, the pixel intensities of the region highlighted in Fig. 5a, were histogrammed, resulting in two Gaussian distributions. The figure of merit (FoM) was defined as:

2 $$\begin{aligned} FoM = \frac{\mu _1 - \mu _2}{\sigma _1 + \sigma _2}~~, \end{aligned}$$

with $\mu _1$ and $\mu _2$ being the means of the two pixel intensity distributions corresponding to open beam and area covered by tungsten with $\sigma _1$ and $\sigma _2$ their standard deviations, respectively. $\sigma$ and $\mu$ were determined by fitting the sum of two Gaussians to the pixel intensity histogram, an example is shown in Fig. 6b. Hence, the FoM optimizes the contrast between tungsten and open beam. The parameter-set $k= 1, m = 1, p = 4, t = 100$, resulting in the highest FoM = 7.91, was chosen for processing all data presented in this work. The corresponding radiograph is shown in Fig. 5b and is visibly cleaner than Fig. 5a. Moreover, the resolution for the chosen data reduction parameter set is with FWHM = 4.37 px within $\sim 20\%$ of the best resolution FWHM = 3.62 px, unlike for the contrast where the FoM = 2.29 for the best resolution parameter set is not even a third of the FoM of the selected parameter set. This confirms that for the present experimental setup optimization for contrast produces best results with acceptable loss of resolution.

### Mock up drum radiography

The radiographs of the samples with the dry and semi-wet resin as well as the steel drum flatfield were recorded in 140 min each while the measurement of the sample with the fully wet resin ran for 480 min. All data were processed into 512$\times$512 pixel TIFF stacks using the parameters and procedure described in “Data analysis setup”. After creating the TIFF stacks in which each slice corresponds to a time-of-flight bin with a width of 1.5625 ns, all slices corresponding to neutron energies between 2.5 MeV to 8 MeV were integrated to retrieve the radiographs. This energy range was chosen because lower-energy neutrons are predominantly scattered, and neutrons above 8-10 MeV produce longer proton tracks and exhibit reduced detection efficiency due to a decreasing elastic cross-section and competing reactions. The energy-resolved nature of our detection system allows us to optimize the integrated energy range to effectively discriminate against these background contributions. Radiographs from this energy range were found to result in the highest contrast for this setup. Frame overlap was neglected in this experiment, as it was found to be less than 2% of the signal at any point in the spectrum. Subsequently, all radiographs were normalized by the integrated proton beam current on the WNR target during their acquisition. To enhance the image quality of the radiographs, to remove the pattern of the scintillator nanoguide matrix, and to determine the transmission through the sample, all images with sample were divided by the steel drum flatfield (also normalized by its corresponding proton current). Such a procedure is also feasible for actual waste drums because the waste containers are standardized and it is possible to obtain a flatfield measurement of such an empty container.

### Mock up drum tomography

For the tomography the setup and sample described in “Experimental setup” were used with the exception that the melamine container was rotated $40^\circ$ around the tomography rotation axis to avoid having all material containers parallel. The tomography was recorded using the resin container with the highest water content, from now on called wet resin. To be able to fully reconstruct the sample, 59 projections were recorded and the sample was rotated $11^\circ$ between every projection. Thus the recorded angles range from $0^\circ$ to $649^\circ$. Data for every projection was collected in 10 min, resulting in a total beam time of 10 h. Due to beam time limitations, a flatfield measurement of the empty steel drum was done in 45 min for a single orientation only.

Before the reconstruction, the projection and the flatfield data were analyzed with the optimal parameters found in “Data analysis setup” and 512$\times$512 pixel radiographs were generated including only events corresponding to neutron energies between 2.5 MeV to 8 MeV similar to “Mock up drum radiography”. Every projection radiograph was then normalized by the flatfield image of the empty steel drum and all pixel outliers exceeding $\times$50 their neighboring intensity values and not a number values (NaNs) were removed using *ImageJ*^52^. From the results the tomography was reconstructed using *Tomviz*^53^, a tomography reconstruction software developed by a collaboration of Kitware, University of Michigan, and Cornell University for electron microscopy. In *Tomviz* the image quality was improved further using the *Remove bad pixels* and *Gaussian filter* functions before running the *ART backpropagation* with 4 iterations (which took about 1.5 h on a Windows laptop with an Intel i5 8th Gen and 8 GB RAM).

### Neutron spectroscopy

Utilizing the detector’s capabilities to record the time-of-flight of the incident neutrons, the energy-dependent transmission spectra of the melamine, the water, and the ion exchange resin were analyzed. For this purpose, the temporal profiles of the transmissions were determined with the help of *ImageJ*’s *Plot Z-axis Profile* function for the respective regions-of-interest covered by these materials. This resulted in transmission over time-of-arrival in the detector’s clock time with a temporal bin width of 1.5625 ns corresponding to the LumaCam’s resolution (c.f. “Data analysis setup”). The time-of-arrival was converted to energy via the relativistic conversion formula:

3 $$\begin{aligned} E(i) = \frac{m_{\text {0,n}} c^2}{\sqrt{1 - \left( \frac{L}{ct_{\text {tof}}(i)} \right) ^2} } - m_{\text {0,n}} c^2~~. \end{aligned}$$

Here, *L* is the distance from the source (spallation target) to the detector, $m_{\text {0,n}}$ is the rest mass of neutrons, *c* refers to the speed of light, and $t_{\text {tof}}(i)$ is the time-of-flight corresponding to the *i*th time bin. With the index $i_{\gamma }$ and the time-of-flight $t_{\text {tof},\gamma }$ of the gamma flash, the neutrons’ time-of-flight was determined as $t_{\text {tof}}(i) = (i - i_{\gamma })\,\times \,{1.5625}\,{ns} + t_{\text {tof},\gamma }$. After conversion, a constant background *B*, which was refined in the fitting process described below, was subtracted from the material and the corresponding flatfield spectra. Afterward, the spectrum behind material was divided by the flatfield spectrum retrieved by applying the same region-of-interest to the flatfield (empty steeldrum) data. The result was normalized by the ratio of the integrated proton currents during the sample and flatfield measurement to receive the absolute transmission. Uncertainties in transmission were estimated from the statistical uncertainties of the count numbers *N* (after background subtraction) by applying Gaussian error propagation. Furthermore, the resulting spectra were corrected for higher energy neutrons being scattered and detected at a time-of-flight corresponding to a lower energy (downscattering), since otherwise the spectra were overestimating the lower energy part. This scattering was likely caused by the sample, as some of the materials contain significant amounts of hydrogen. To correct for this, a flatfield corrected spectrum of an area without sample above the melamine and water containers was used as a second, energy-dependent normalization for the material spectra. No detection efficiency response function was applied, as all time bins were normalized with (divided by) their respective flatfield bin. Because any energy-dependent detector response affects the flatfield and the sample measurement equally, it cancels out when those two are divided.

To validate all spectra, they were compared to a theoretical transmission model (the transmissions calculated and fitted to the data in the way described here will be referred to as theoretical (model) transmissions, whereas transmissions determined from Monte-Carlo simulations will be referred to as simulated transmissions) $T(E_n)$ calculated using energy-dependent total cross-sections $\sigma _i(E_n)$ obtained from JENDL-4.0^25^ in Lambert-Beer’s law:

4 $$\begin{aligned} T(E_n) = \tfrac{I(E_n)}{I_0(E_n)}= R(E_n)*e^{-\Sigma _{i=1}^k \sigma _i(E_n) n_i d}. \end{aligned}$$

Equation 4 gives the energy-dependent intensity $I(E_n)$ behind a material with thickness *d* consisting of *k* different elements with respective atomic densities $n_i$ (and energy-dependent cross-sections $\sigma _i(E_n)$). Because the temporal, and hence also the energy resolution of the experimental data is far below the resolution of the JENDL-4.0 cross-sections, the theoretical curve has been convoluted with a response function $R(E_n)$ that is represented by an exponentially modified Gaussian distribution function^54^:

5 $$\begin{aligned} f(x) = \frac{1}{2K \tau } \exp \left( \frac{1}{2K^2} - \frac{x - x_0}{K\tau } \right) \text {erfc} \left( \frac{\frac{1}{K} - \frac{x - x_0}{\tau }}{\sqrt{2}} \right) ~~. \end{aligned}$$

Equation 5 is a Gaussian with an additional exponential part to account for effects that are asymmetric in time like neutron scattering. In contrast to the Gaussian pulse coming from the spallation target^55^, the asymmetric pulse shape required to interpret the measured transmission data provides further evidence that downscattering is occurring as a result of the moderating materials present in the beam, such as water and iron. This is similar to the asymmetric pulse shape emitted from a water moderator for a pulsed thermal and epi-thermal neutron source. The parameter *K* adjusts the ratio between Gaussian and exponential decay (the function converges to a Gaussian in the limit of ${K \rightarrow 0}$ ), $x_0$ is the mean of the distribution, and $\tau$ is a scaling parameter determining the width of the distribution. The transmission model was fitted to the experimental transmission data of the water, melamine, and ion exchange resin independently using a least-squares method. In this fitting process *K*, $x_0$, $\tau$, the material density $\rho$ and the background *B* were varied.

Lastly, the entire image stack of the measurement with the wet sample was analyzed to determine where water and melamine were located in the sample. For this purpose, ${16}\,{\hbox {px}}\,\times \,{16}\,{\hbox {px}}$ superpixels were formed, the spectra of which were then determined using the method described above. Each of these spectra was then fitted with the following model:

$$\begin{aligned}&a \times {\text {best}}\_{\text {fit}}(melamine) + b \times {\text {best}}\_{\text {fit}}(water) +\\ &c \times {\text {best}}\_{\text {fit}}(resin) + d ~~, \end{aligned}$$

where *a*, *b*, *c* and *d* are the scaling parameters varied and *best_fit* denotes the theoretical transmission of the respective material which has been fitted to the material data before, as described in the previous paragraph. For a superpixel behind one of the materials of interest, the corresponding *best_fit* should have an increased contribution (higher scaling parameter) to the fitted model compared to pixels without that material. Therefore, the resulting parameter triples (*a*, *b*, *c*) are indicators of the abundance/likelihood of melamine, water, or ion exchange resin in front of the corresponding superpixel. Hence, a material map can be generated from them by applying thresholds.

### PHITS simulations

The Monte Carlo code PHITS^37,56^ version 3.31, developed and maintained by the Japan Atomic Energy Agency, is used to compare and verify the experimentally obtained results. For neutron transport, the JENDL-4.0^25,33^ nuclear data library is used. The source is based on the measured 60R neutron spectrum published by Devlin *et al.*^55^. The time distribution of the neutron pulse follows a Gaussian distribution with a FWHM of 1 ns. The beam spot diameter is set to 3 inches. The material sizes, shapes, and arrangement are the same as for the actual sample described in “Experimental setup”, except that all containers are fully filled with their theoretical density for each material, which was not possible in practice.

At the position of the detector a *[T-Track]* tally is used to score the 2D neutron distribution according to its time-of-flight in a time range that corresponds to the energy range of 2 MeV to 8 MeV. Each time bin has a width of 1.5 ns. The total detector area in the simulations is ${10}\,{\hbox {cm}}\,\times \,{10}\,{\hbox {cm}}$ and each pixel has an area of ${0.5}\,{\hbox {mm}}\,\times \,{0.5}\,{\hbox {mm}}$. During each simulation, a total of $10^{9}$ neutrons were transported.

## Results and discussion

### Radiography

**Figure 7:**
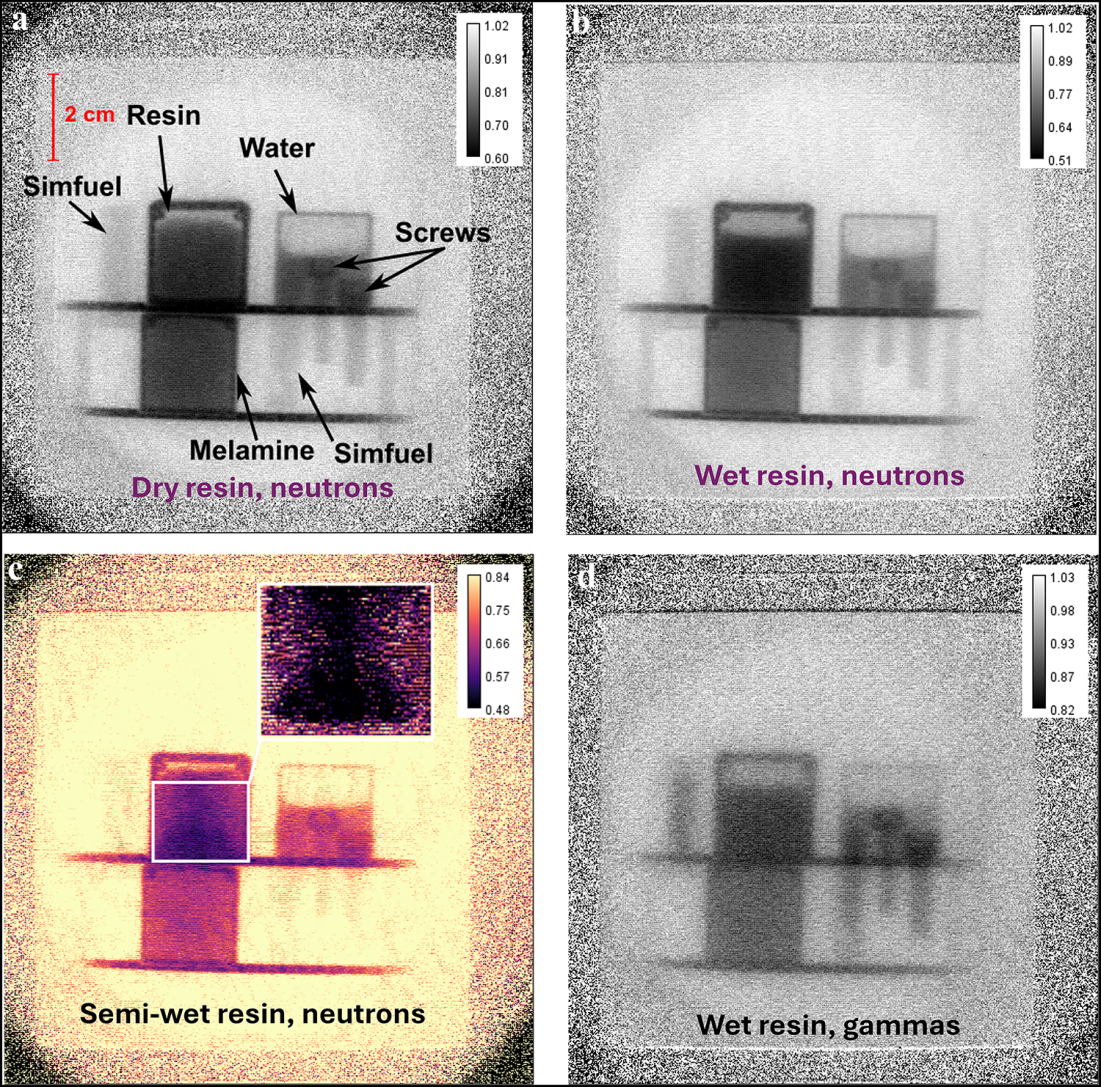
Radiographs of the sample described in “Experimental setup” (**a**) with dry, (**b**) wet, and (**c**) semi-wet ion exchange resin, made with 2.5–8 MeV neutrons. A radiograph of the wet resin setup made with gammas is shown in d). The enlarged window in (**c**) shows the difference image of the semi-wet and the dry resin (normalized semi-wet - normalized dry) for the region of interest marked white.

Figure 7a shows a radiograph of the sample containing the dry resin. All objects of the sample depicted in Fig. 4 are visible, even the 2 mm radius legs of the sample holder table. Furthermore, the fill levels of the resin in the upper left container and the water in the upper right container are clearly discernible to a degree of precision allowing to see the concave surface behavior of the water due to adhesion. Comparing Fig. 7a and 7b, showing the dry and fully wet resin, respectively, the difference in attenuation is immediately visible. For example in the case of the dry resin the container wall and resin show different attenuations while for the wet resin the container wall and resin show much less difference in attenuation.

Table 1 shows averaged transmissions for areas covered by different materials from the experiment and simulation with their estimated standard deviations. For most materials the *PHITS* simulations and experimental transmission values are in agreement within the determined uncertainties, benchmarking the ability to simulate setups such as described here with *PHITS*. The transmission values for wet resin (container with resin completely filled with water) and dry resin differ by more than their uncertainties. The semi-wet resin shows an non homogeneous distribution of water, leading to a larger estimated standard deviation. However, the average value lays between wet and dry resin containers. Hence, the wet resin can be distinguished from the dry resin due to the significant difference in transmission. Figure 7c shows the radiograph of the sample with the semi-wet resin in a magma color scheme to enhance the visible contrast in the resin container. The enlarged window depicts the subtraction of the normalized (by proton current) image of the dry resin from the normalized image of the semi-wet resin. For the semi-wet sample, it can be seen in the radiograph and even better in the difference window, that the water was poured into the container from the middle, resulting in a higher water content in the middle region. Furthermore, it becomes obvious that most of the water is collected at the bottom of the container. The transmission value of the wet ion exchange resin is slightly above the simulation result. This is likely due to the uncertainty in the resin’s density because not all of the container’s volume is occupied. More importantly, the simulated difference in transmission between the wet and the dry resin ${\bar{T}}_{\text {diff}} = 0.14 \pm 0.01$ matches the measured difference ${\bar{T}}_{\text {diff}} = 0.11 \pm 0.03$ within the limits of the uncertainties, proving that the abundance of water can be correctly reproduced. The slight discrepancy of 7.2% between the experimental and the simulated transmission of melamine is presumably caused by an issue with the weight of the melamine sample, as discussed further in “Neutron spectroscopy”. Lastly, it is worth noting that the (integrated) transmission of melamine is indistinguishable from the transmission of water and dry resin within the uncertainties, highlighting the importance of spectroscopic (energy-dependent) characterization techniques to recognize different materials.

**Table 1:** Experimental and simulated transmissions with uncertainties with a covering factor of 1.

Material	Experimental T	Simulated T
Dry resin	$0.67 \pm 0.02$	$0.64 \pm 0.01$
Wet resin	$0.56 \pm 0.02$	$0.50 \pm 0.01$
Semi-wet resin	$0.60 \pm 0.04$	-
Water	$0.71 \pm 0.02$	$0.69 \pm 0.02$
Screws	$0.88 \pm 0.03$	$0.91 \pm 0.02$
Fuel rods	$0.90 \pm 0.03$	$0.88 \pm 0.02$
Melamine	$0.69 \pm 0.02$	$0.64 \pm 0.01$

Unlike Fig. 7a–c showing radiographs made with 2.5–8 MeV neutrons, Fig. 7d depicts a radiograph of the sample with the wet resin made with photons from the gamma flash also coming from the tungsten spallation target. Thanks to the event-mode capabilities of the Timepix3-based detector employed in this work, both neutrons and gammas can be measured simultaneously and distinguished due to their difference in time-of-flight. Comparing the gamma radiograph to the neutron radiograph in Fig. 7b, the first striking difference is the lower resolution in the gamma image, possibly due to the gammas being scattered and attenuated more in the steel drum surrounding the sample. Moreover, the contrast of the lower density samples is inferior and details like the adhesion of the water and the difference between the melamine and its container vanish. However, materials with higher density, like the steel screws and especially the heavier elements inside the SIMFUEL rods, are much better visible in the gamma radiograph. Therefore, collecting neutron and gamma radiographs of the sample delivering complementary information facilitates an improved material identification as demonstrated by Kumar et al.^57^. This highlights the benefits of the unique detector system employed here in combination with a high-resolution short-pulse MeV neutron source enabling multi-modal analysis of the sample.

### Tomography

**Figure 8:**
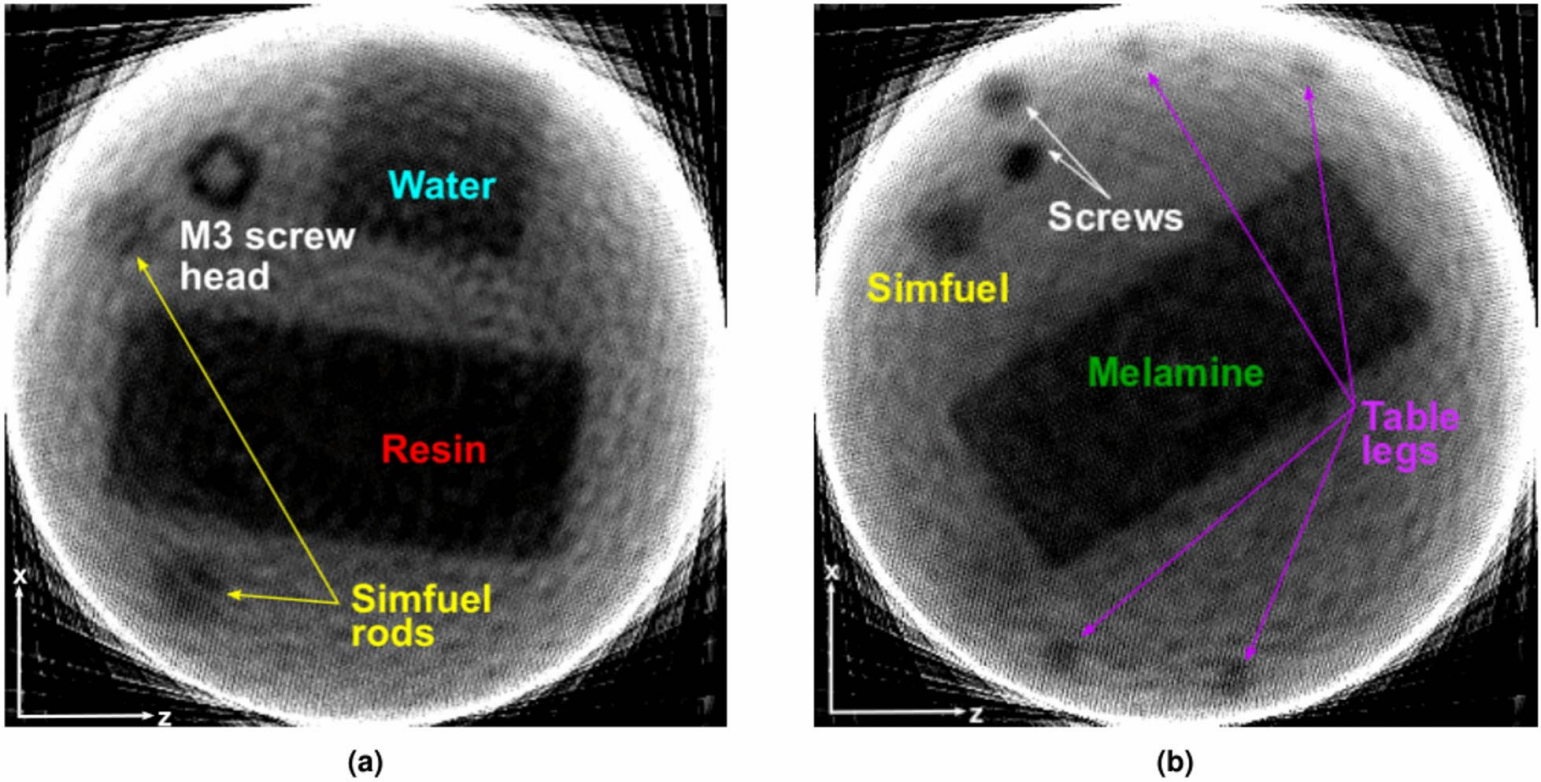
Reconstructed tomography slices in the horizontal plane for two different heights (**a**) Slice of the upper part of the sample assembly showing the wet resin container, the water container, two SIMFUEL rods, and the head of the M3 screw (the second screw starts below this slice as it is lower). The M3 head has a diameter of 5.68 mm with the largest diameter of the hole being 2.58 mm. (**b**) Slice of the lower part of the sample assembly showing the melamine container, two SIMFUEL rods and both screws. Even the legs of the upper sample table are visible.

Figure 8a shows one out of 306 slices in the horizontal xz-plane of the reconstructed sample (the neutrons travel in the z-direction and xy is the radiograph plane). All objects present at the corresponding height of this slice are visible. The resin and the water containers are resolved with clean edges, although the outer rim of the water container fades into a bright reconstruction artifact resulting from the neutron beam being slightly smaller than the sample assembly in some orientations. Both SIMFUEL rods are detectable and even the hole in the hex head of the M3 screw is distinctly visible.

In Fig. 8b, a slice of the lower part of the sample is depicted. From this slice it is obvious that the melamine container has been rotated $40^{\circ }$ against the water and resin container orientations. Furthermore, both screws can be seen in the upper left corner of the image and the SIMFUEL rod going down to the lower sample holder floor is visible too. The other SIMFUEL rod is not shown in this slice, as the rod does not go down that far in the sample (compare Fig. 4). Additionally, even the four 4 mm in diameter legs of the upper sample holder table can be recognized at the edge of the neutron beam during measurement. The distance between the two legs (center to center) calculated from the tomography result is $D=({2.00 \pm 0.02})\,{\hbox {cm}}$ which matches the actual distance $D= ({2.00 \pm 0.01})\,{\hbox {cm}}$ exactly, thus proving that plastic objects of mm size can be detected through thick steel shielding and their shape and position within the sample can be reconstructed precisely using MeV neutrons.

### Neutron spectroscopy

**Figure 9:**
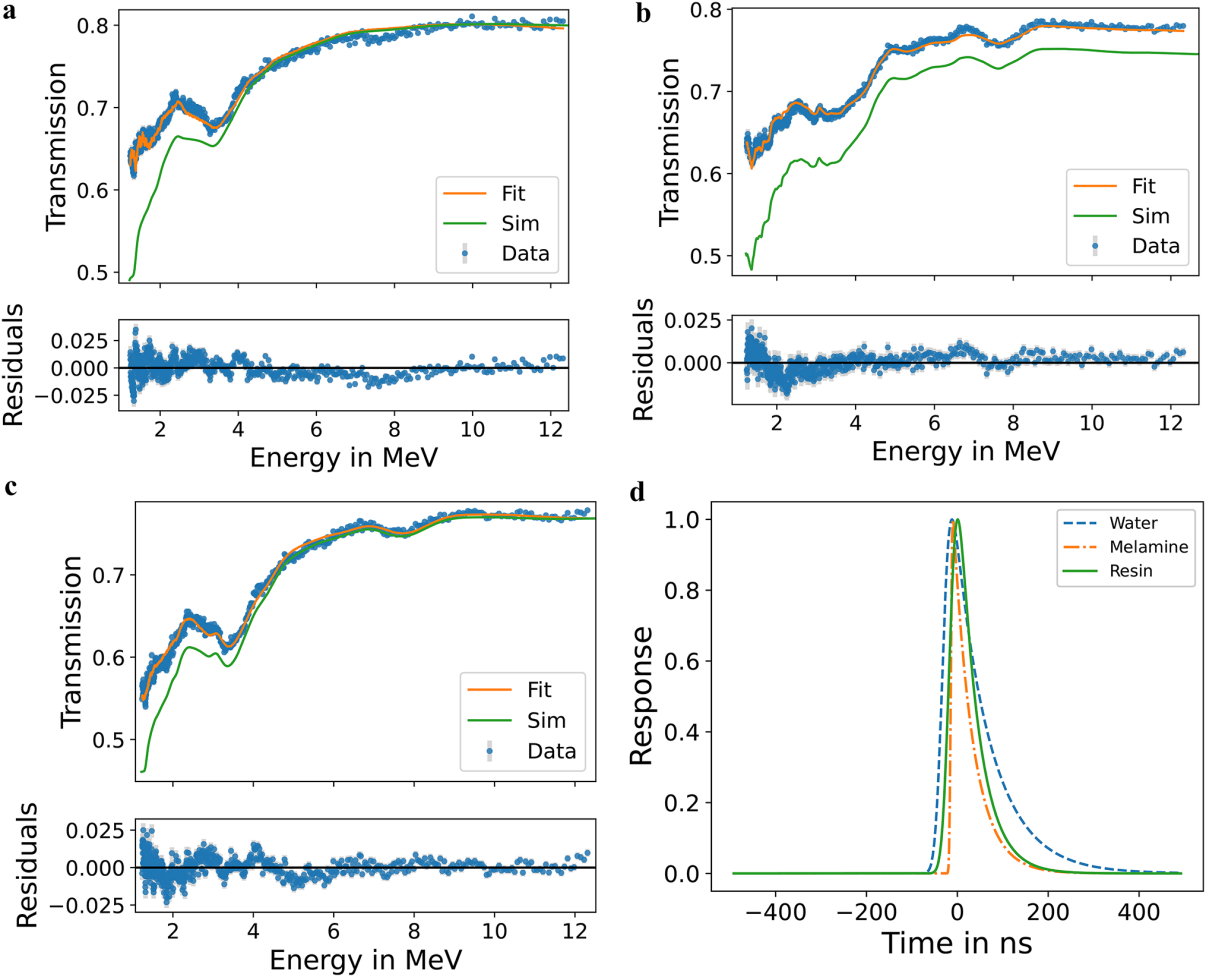
Neutron transmission vs. neutron energy behind water (**a**), melamine (**b**), or dry ion exchange resin (**c**). Experimental data is shown in blue while the best fit of the theoretical transmission, obtained as described in “Neutron spectroscopy”, is shown in orange. The *PHITS* simulations are plotted in green. The difference between experiment and best fit is shown above. (**c**) The temporal responses determined during the fitting procedure for each material.

Figure 9 shows the neutron spectra measured behind water, melamine, and dry resin with the best model fit to equation 4 as described in “Neutron spectroscopy” as well as the *PHITS* simulations. Generally, features in the theoretical model and the simulation agree with the experimental transmission spectra. For the water data, the strong resonance features between 2 MeV to 4 MeV caused from neutron attenuation by oxygen in the water are visible, allowing to distinguish water from other materials without oxygen such as melamine. The fitted parameters and their associated uncertainties determined by *lmfit*^58^ are displayed in Table 2 for water, melamine, and the ion exchange resin.

According to the fit of the corresponding neutron transmission spectra, the sample density of the 2 cm water layer was $\rho =({1.080 \pm 0.002}){\hbox {g}/\hbox {cm}^3}$ which is $\sim 8\%$ above the expected density of $\rho ={0.998 \pm 0.000}{\hbox {g}/\hbox {cm}^3}$ for water at room temperature^59^. This can be explained with the contribution from the 2 mm plastic container layer to the experimental data, which was neglected in the theoretical model. Likely, the carbon inside the plastic of the container also caused the small dip around 7.7 MeV where the model is slightly overestimating the data, as there is a $\sim {1}\,$MeV wide carbon resonance around 7.8 MeV. The fitted density of the 5 cm layer of dry resin $\rho =({0.553 \pm 0.001}){\hbox {g}/\hbox {cm}^3}$ matches the actual density of $\rho =({0.569 \pm 0.028}){\hbox {g}/\hbox {cm}^3}$ within the uncertainties, again not considering the attenuation caused by the container in the theoretical model. For the melamine the fitted density $\rho =({0.609 \pm 0.001}){\hbox {g}/\hbox {cm}^3}$ underestimates the measured (weighed) density $\rho =({0.729 \pm 0.055}){\hbox {g}/\hbox {cm}^3}$ significantly, as was already to be expected by the mismatch in measured and simulated transmission (c.f. “Radiography”). Notably, the width of the response function $\tau$ and the background *B* of the fit to the water data are significantly larger compared to the melamine and resin results. This is consistent with the water sample being essentially a moderator for the neutrons. The temporal response functions determined during the fitting procedure for each material are presented in Fig. 9d. They are broader than in previous experiments conducted with the same setup, and the reason for this remains under investigation. Nevertheless, this study demonstrates that the materials under investigation can also be identified with this reduced resolution. In Fig. 9b the fitted spectrum of melamine is shown, reflecting the lower background and sharper temporal response indicated by the parameters in Table 2. The agreement between measured data and theory is very good with only one peak at 6.8 MeV slightly overestimated by the experimental data. All expected features from nitrogen and carbon are reproduced by the measured spectrum and the $\chi ^2=2.49$ of the fit is significantly better than the $\chi ^2=5.06$ of the water fit. The spectrum of the dry ion exchange resin is fitted in Fig. 9c, again showing good agreement between experiment and fit ($\chi ^2=3.74$), despite the composition of the resin being estimated from information from the material safety data sheet which may deviate from the proprietary actual composition.

The *PHITS* simulations of the energy-dependent transmission spectra, using the mass and volume measured from the actual sample materials to calculate the density, generally agree well with the experimental results. In particular, the structure in the transmission curves resulting from the resonance features of the cross-sections, are well reproduced. The main discrepancies for all three materials are at the lower energies, potentially due to issues with background subtraction to calculate the transmission signal or incorrect modeling of the inelastic neutron interaction with the iron in the drum. The observed offset between simulation and experimental data for melamine could be explained by problems with the measured mass of the sample being $\sim 20\%$ too heavy. The sample mass was determined several months after the neutron experiment, allowing for the melamine to adsorb water from air humidity, resulting in mass increase^60^.

**Figure 10:**
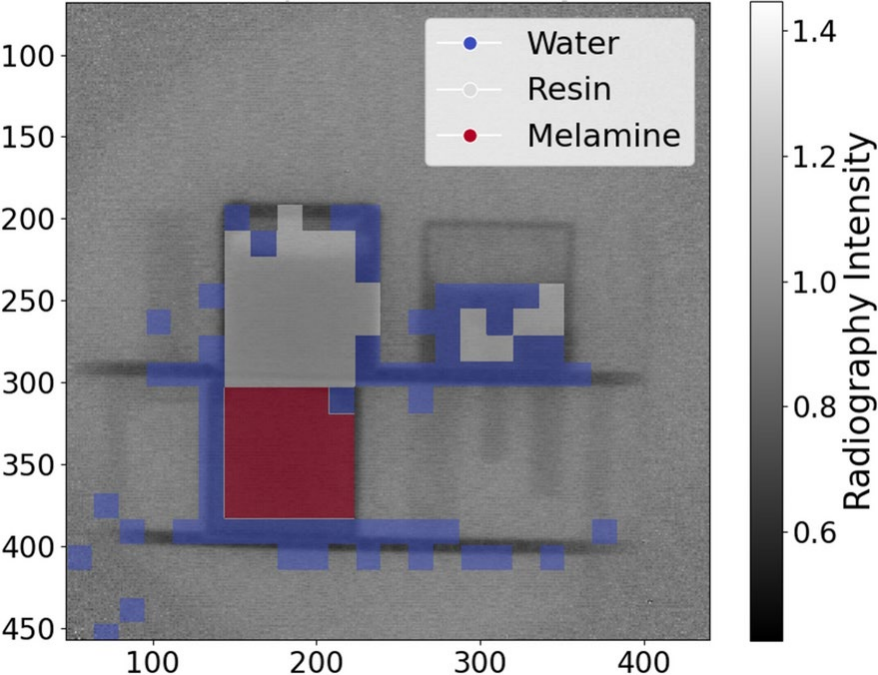
Material map showing where water, melamine, and the ion exchange resin are abundant in the sample according to the evaluation method described in “Neutron spectroscopy”.

Finally, the mapping of water, melamine, and the resin in the sample is shown in Fig. 10 (c.f. “Neutron spectroscopy” for details on the localization procedure). While melamine is detected very reliably only at the material position of melamine, some superpixels at locations of the 3D printed plastic are misidentified as water. This is likely due to the oxygen present in the PETG compound used for the 3D printing confusing the cross-section based material identification algorithm. Moreover, some superpixels inside the water sample are mistakenly identified as ion exchange resin as a result of the similarity of their spectra (compare Fig. 9a and b). However, the rest of the water sample is recognized as water and the resin sample is correctly identified as such. Overall, this experiment is a first demonstration of the potential capability to recognize and localize materials made from light elements such as water, melamine, and ion exchange resin in a nuclear waste package using energy-resolved MeV neutron imaging techniques. This, in turn, could enable the detection of residual liquids and potential toxins.

**Figure 11:**
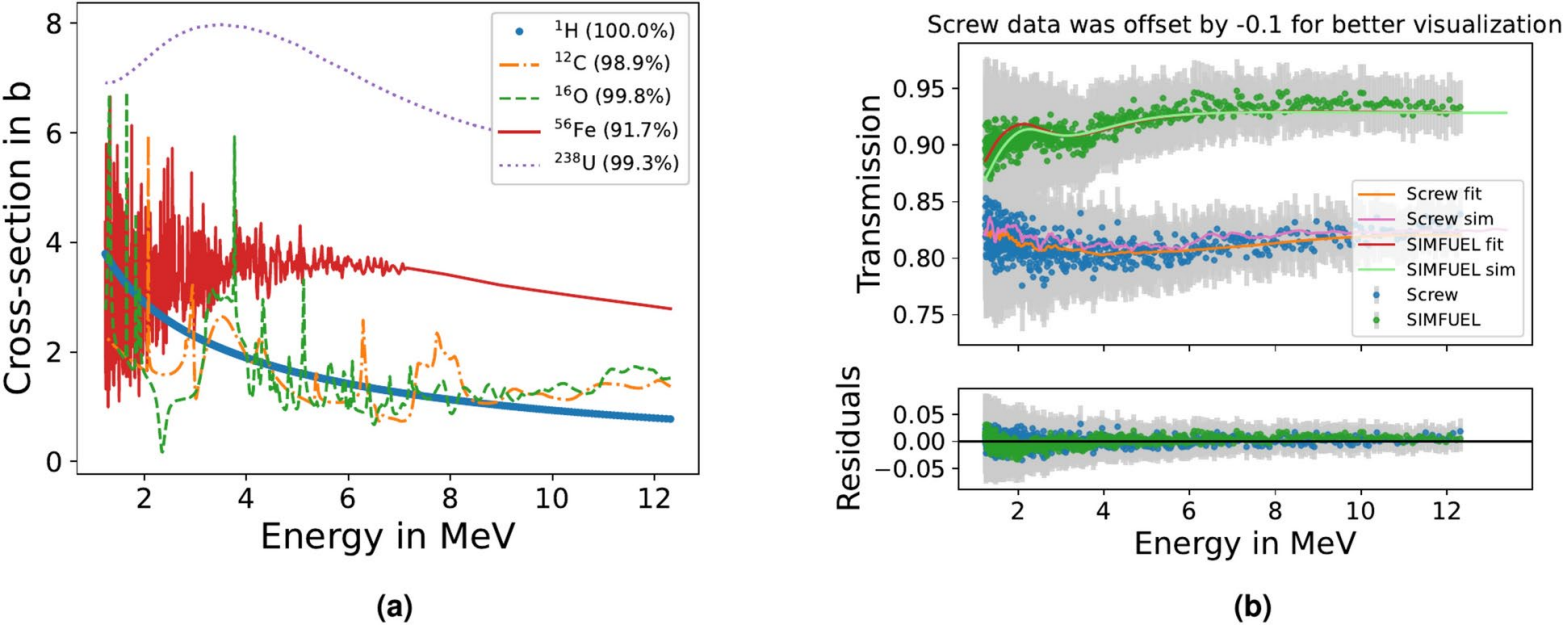
(**a**) Comparison of total cross-sections of MeV neutrons for some relevant elements. The natural abundance of the respective isotopes is shown next to the element symbol in the legend. Data taken from^25^. (**b**) Transmission spectra of the M4 screw and the upper left SIMFUEL rod together with the corresponding fits and simulation results. For better visualization, the screw data, simulation results, and fit have been subtracted with 0.1. To optimize the matching of simulation and data, SIMFUEL simulation results were multiplied with 1.036.

The screws and SIMFUEL have been excluded from the mapping procedure, because their spectra are harder to identify as they lack prominent features. Figure 11a compares the total cross-sections of MeV neutrons for some elements relevant to this work. While light elements such as carbon and oxygen exhibit the prominent, wide features used for the material mapping above, heavy elements such as iron or uranium have a smooth cross-section or unresolvable resonance structures. Hydrogen also has a smooth cross-section which is why with MeV neutrons water is identified via the oxygen it contains, not the hydrogen as is the case for cold or thermal neutrons. Despite the lack of prominent features, heavier materials such as SIMFUEL and steel could be distinguished using more comprehensive analysis in the future. The transmission spectra of the M4 screw and the upper left SIMFUEL are compared in Fig. 11b showing their different shapes. While the spectrum of the screw bends upwards, the SIMFUEL spectrum is bent downwards and shows a small resonance feature around 2 MeV reflecting the oxygen it contains. To fit the SIMFUEL data, a $v ^{238}\text {U} + (1-v) ^{16}\text {O}$ composition was assumed, and $v$ was optimized together with the other parameters of the *lmfit* model. The result $v= 0.19 \pm 0.03$ underestimates the expected value of 0.33 slightly, but considering that all other oxides have been neglected, it is sufficiently close for a first approximation. This composition analysis methodology will be further explored and refined in future studies. Combined with an analysis of the gamma transmission, these distinct neutron transmission spectra should also allow the identification of heavy materials^57^. While in this experiment gamma radiographs were recorded based on the time-of-flight, for quantitative analysis of the gamma transmission the energy spectrum of the gamma radiation would need to be characterized. Whereas fission gammas, as used at NECTAR by Kumar *et al.*, are reasonably well described by a 1 MeV average energy, the energy spectrum of gammas emitted during spallation induced by 800 MeV protons is more complex and therefore analysis of the gamma radiographs was beyond the scope of the present article. However, in future studies the dual-mode detection capabilities of our detector system could represent a major advancement for comprehensive analysis of the light and heavy materials in nuclear waste packages. While traditionally two detector systems often requiring separate exposures would be necessary to map neutron and gamma transmissions, the setup presented in this work could do both simultaneously with a single detector system.

**Table 2:** Fit results for water and melamine.

	Water	Melamine	Resin
*D*	$({1.080 \pm 0.002}){\hbox {g}/\hbox {cm}^3}$	$({0.609 \pm 0.001}){\hbox {g}/\hbox {cm}^3}$	$({0.553 \pm 0.001}){\hbox {g}/\hbox {cm}^3}$
*K*	$7.8 \pm 2.2$	$18.7 \pm 18.1$	$3.35 \pm 0.43$
$x_0$	$({918.3 \pm 1.2})\,{ns}$	$({935.2 \pm 0.7})\,{ns}$	$({934.3 \pm 1.0})\,{ns}$
$\tau$	$({12.7 \pm 2.6})\,{ns}$	$({2.3 \pm 2.2})\,{ns}$	$({12.7 \pm 1.4})\,{ns}$
*B*	$1545.8 \pm 9.5$	$720.7 \pm 9.3$	$685.9 \pm 10.1$
$\chi ^2$	5.06	2.49	3.74

Parameters are introduced and explained in “Neutron spectroscopy”.

## Conclusion and outlook

The research presented in this study shows that materials consisting of light elements such as water, melamine, or resin, can be identified inside a 2.54  cm wall thickness steel drum by energy-resolved MeV neutron radiography. Denser materials inside the steel drum, such as nuclear fuels or metal objects, exhibited less spectral features, necessitating more comprehensive analysis for their identification. Other radiography probes, such as X-rays or thermal/epi-thermal neutrons, are unable to identify the light materials or penetrate the walls of the steel drum, respectively. In particular, this work marks an advancement in the field of nuclear waste characterization, demonstrating the potential of energy-resolved MeV neutron radiography as a powerful tool for non-destructive evaluation of nuclear waste packages, addressing critical challenges in nuclear waste management. Key findings from the study include the visualization of different objects such as steel screws, SIMFUEL rodlets, and 3D printed table legs, as well as the localization of water, ion exchange resin, and melamine within the steel drum. The materials could be resolved in a simple radiography, but it was also possible to reconstruct their shape and location in 3D using tomographic reconstruction, allowing e.g. to see the hole in the head of an M3 screw (c.f. Fig. 8a), and to determine the distance between the 4 mm diameter plastic table legs with an accuracy of ${200}\,\upmu {\hbox {m}}$. Furthermore, in Sect. 3.1 it was demonstrated that residual water in ion exchange resin can be detected, as the dry, semi-wet, and wet samples had significantly different transmissions (Fig. 7c). This is crucial for the characterization of nuclear waste because spent ion exchange resin from filters for liquid radioactive waste streams is a substantial part of the waste and knowledge of the moisture content is crucial for transport and storage^34–36^. The experimentally measured transmission spectra were validated by Monte-Carlo simulations of the experiment using *PHITS*. The overall agreement between simulated and measured data (c.f. “Radiography”) confirmed the effectiveness of the methodology in accurately assessing the samples inside the mock up waste package. The benchmarking of the simulations with experimental data establishes our experimental setup as suitable for future benchmarking as well as lending credibility to simulations of hazardous materials more difficult to assess experimentally. The energy-resolving capabilities of short-pulse MeV neutron sources and the detector system used here revealed the internal structure of the mock up waste drum, highlighting the capability to distinguish materials based on their energy-dependent neutron cross-section. The energy-resolved neutron transmission spectra of water, melamine, and ion exchange resin were fitted to and compared with theoretical transmission spectra derived from JENDL-4.0 cross-section data (c.f. “Neutron spectroscopy” and “Neutron spectroscopy”), demonstrating very good agreement of experiment and theory. In all analyzed spectra the prominent cross-section features of oxygen (in the case of water and resin), and nitrogen and carbon (melamine and resin) are observable and match the expected features in shape, position, and size. The application of neutron spectroscopic analysis between 1 MeV to 12 MeV (c.f. Fig. 10) further enhanced the nuclear waste characterization process by enabling the localization of different materials based on cross-sections calculated for known chemical compositions. While some superpixels were misidentified as water, likely due to the oxygen in the PETG plastic present in those superpixels, the water sample could be localized correctly by this method. Although some superpixels of the water were identified as resin because of their similar spectral shapes (compare Fig. 9a and c), the resin and melamine materials could be correctly localized, highlighting the capabilities of this setup to analyze nuclear waste packages more elaborately than previous radiography experiments^16^.

Looking forward, future research directions include the continued refinement of detector technology and analysis methods to improve spatial and energy resolution capabilities as well as material identification. Larger, and more complex mock up waste packages up to full-sized real waste drums will need to be analyzed and blind tests will have to be conducted to resemble actual waste package characterization more accurately. Additionally, the integration of artificial intelligence and machine learning algorithms for automated data analysis and real-time decision-making could further enhance the efficiency and reliability of energy-resolved MeV neutron analysis in nuclear waste management. Moreover, it should be mentioned that significant resources have been utilized for this study, as LANSCE is neither a small nor a cheap source, and hence future research also must aim to provide and utilize more affordable and compact short-pulse (<1 ns pulse width) neutron sources. A promising candidate in the future are laser-driven neutron sources as their short pulse width can be beneficial in this case^17–19^. In summary, this study represents a significant step forward in the field of nuclear waste characterization, demonstrating the feasibility and efficacy of energy-resolved MeV neutron analysis as a powerful tool in non-destructive evaluation of nuclear waste packages. These first proof-of-concept results contribute to advancing scientific understanding and technological innovation in nuclear waste management, with broader implications for environmental protection and public safety.

## Data Availability

Data used for this work is available on request. If You wish to request the data, please contact sven@lanl.gov.
